# Improved leaf area index estimation in Jujube trees by fusing spatial-temporal features from UAV RGB time-series with a deep learning framework

**DOI:** 10.3389/fpls.2026.1798751

**Published:** 2026-05-18

**Authors:** Yaxing Liu, Yonglin Gao, Jianting Wang, Dongdong Liu, Zhong Zheng

**Affiliations:** 1 Agricultural College, Shihezi University, Shihezi, China; 2 Xinjiang Shidaguoli Agricultural Science and Technology Co., Ltd, Alaer, China

**Keywords:** CNN-GRU, deep learning, drone remote sensing, leaf area index, precision agriculture

## Abstract

To achieve high-precision and high-efficiency estimation of the Leaf Area Index (LAI) of jujube trees using drone remote sensing, and to overcome the limitations of traditional vegetation index methods, such as saturation in the later stages of crop growth, sensitivity to background noise, and difficulty in capturing temporal dynamics, this study proposes a parallel hybrid deep learning framework using CNN-GRU. The model adaptively extracts spatial-spectral local features from drone RGB images through a convolutional neural network (CNN) branch, while a gated recurrent unit (GRU) branch learns the sequential evolution of LAI during key phenological periods. Finally, a meta-learner integrates spatial-temporal information for decision-making. To verify the model’s effectiveness and prediction performance, the study systematically collected multi-temporal ground-measured LAI data and synchronized drone remote sensing images during critical growth stages of jujube trees in two independent years: 2024 (Bachu, Xinjiang) and 2025 (Alaer, Xinjiang). A series of spectral and texture indices were extracted as model inputs. The experimental results show that the proposed CNN-GRU model exhibits excellent learning and fitting capabilities on the training set, with an *R*^2^ value of 0.839. On the test set, after optimization with data augmentation strategies, the model’s prediction accuracy is significantly improved, with prediction accuracy reaching its best level, with an *R*^2^ of 0.83 and an RMSE of 0.150. All error metrics outperform mainstream comparative models such as Transformer, KNN, MLP, and CNN. This study demonstrates that the hybrid deep learning architecture, combining spatial feature extraction and time-series modeling, is an effective approach for accurate and robust remote sensing inversion of crop LAI in complex agricultural scenarios, providing a reliable technical tool for the digital management of smart orchards and precise agricultural decision-making.

## 1 Introduction

The Leaf Area Index (LAI) is a key biophysical parameter that measures the total area of green leaves per unit ground area (Fang et al., 2019), directly relating to vegetation photosynthesis, transpiration efficiency, carbon-nitrogen cycles, and ultimately productivity (Bonan, 1993; Ben-Asher et al., 2006; Pierce et al., 1994; Burroughs et al., 2023). In precision agriculture systems, rapidly and accurately obtaining crop LAI is crucial for growth diagnosis, yield forecasting, precise water and fertilizer management, and disaster assessment (Rosso et al., 2022; Sun et al., 2024; Shi et al., 2024). Although traditional field measurement methods for LAI provide high accuracy, they are time-consuming, labor-intensive, destructive, and difficult to achieve continuous monitoring at the plot or regional scale (Stenberg et al., 1994). In recent years, unmanned aerial vehicle (UAV) remote sensing, with its high spatial-temporal resolution, flexible flight planning capability, and relatively low operational and equipment cost, has emerged as a transformative technology for agricultural geospatial mapping and vegetation dynamic monitoring. Compared with spaceborne satellite remote sensing, UAV systems can effectively avoid the interference of cloud cover and atmospheric attenuation, achieve customizable high-frequency repeated observations across key phenological periods, and capture centimeter-level high-definition imagery that retains fine structural details of crop canopies; compared with traditional ground-based field surveys, UAVs can realize non-destructive, continuous and full-coverage data acquisition over large-scale planting areas, breaking the spatial limitation of discrete point-scale measurements and supporting the refined mapping of vegetation parameters from plot to regional scales (Dhruwa and Garg, 2025). This technology has opened a new avenue for quickly and non-destructively obtaining canopy information in the field, and has become an important bridge connecting ground-based point measurements and large-scale satellite observations, providing a stable and efficient technical solution for large-scale spatial mapping of crop biophysical parameters and quantitative analysis of canopy spatial heterogeneity in complex orchard and field planting scenarios, greatly promoting crop phenotype research and the development of precision agriculture (Wu et al., 2023; Gao et al., 2025; Bouguettaya et al., 2022).

For a long time, LAI estimation based on optical remote sensing has primarily relied on empirical vegetation indices. Many vegetation indices have been developed to enhance vegetation signals by combining reflectance from different bands, aiming to establish statistical relationships with LAI (Myneni et al., 1995). Kamenova and Dimitrov (2021) systematically evaluated 40 vegetation indices for estimating winter wheat LAI and found that, although some indices performed well, the overall estimation accuracy for LAI was still not ideal. They also noted that separate modeling for different phenological periods was necessary to improve early-stage estimation accuracy. However, these methods have significant limitations. Many widely used vegetation indices tend to saturate during the later stages of crop growth when the canopy is dense, leading to a sharp decline in sensitivity to LAI changes. Bajocco et al. (2022) further pointed out that single indices such as NDVI have poor generalizability when converting to LAI, with the functional form highly dependent on specific environmental conditions and available data, making direct extrapolation difficult. To overcome background (soil, residue) interference, Li et al. (2023) proposed a physically-based residual-soil-adjusted red-edge index (RSARE), which effectively alleviated the impact of background mixing and chlorophyll content during the early growth stage. However, this remains a “patchwork” improvement aimed at specific interferences, and its performance in complex backgrounds and throughout the entire growing season still requires further validation.

To enhance modeling capabilities, machine learning methods have been introduced into LAI estimation. Ma et al. (2021) used extreme learning machines (ELM) combined with spectral transformations and vegetation indices to estimate cotton LAI and achieved good results, demonstrating the advantage of machine learning in handling nonlinear relationships. A comparative study by Chatterjee et al. (2025) further revealed that directly using canopy spectral reflectance as input to machine learning models significantly outperforms vegetation index-based methods in mid-to-late growth stages. This is because reflectance data retains more complete spectral information, avoiding information loss during the vegetation index construction process. However, existing machine learning methods still face two core challenges: First, feature dependence and representation limitations. Whether using vegetation indices or raw reflectance, traditional machine learning models have limited feature extraction capabilities and cannot automatically learn deep, robust feature representations from high-dimensional, spatial-spectral-temporal data Azadbakht et al. (2019). Second, there is insufficient modeling of temporal dynamics. Crop LAI change is a continuous time-series process. Wang et al. (2022) used LSTM networks to process LAI time series for yield prediction, demonstrating the potential of recurrent neural networks in capturing temporal dependencies. However, their study focused on yield prediction rather than LAI estimation itself, and the model input was pre-processed LAI products, not raw remote sensing data, leaving the end-to-end estimation problem from remote sensing data unresolved. The key challenge now is how to develop a deep learning model capable of simultaneously and adaptively mining spatial-spectral features and phenological temporal patterns from remote sensing data, while being suitable for limited sample scenarios, to achieve high-precision and high-robustness LAI estimation.

To address the above deficiencies, this study proposes the development of a parallel hybrid deep learning framework for high-precision and high-generalization remote sensing estimation of jujube tree LAI. We propose a CNN-GRU parallel hybrid network architecture. This model automatically extracts spatial spectral local correlations and deep features sensitive to LAI from UAV remote sensing images through a convolutional neural network (CNN) branch. Simultaneously, a gated recurrent unit (GRU) branch specializes in learning the dynamic changes of LAI during key phenological periods. The abstract features extracted from the two branches are adaptively fused and decided upon in a meta-learner, enabling collaborative utilization of spatial information and temporal context. Additionally, to address the issue of limited labeled samples in agricultural remote sensing, this study introduces targeted data augmentation strategies to enhance the model’s generalization ability. Systematic experiments will be conducted to comprehensively validate the superiority of the proposed CNN-GRU hybrid model in terms of feature correlation, prediction accuracy, model robustness, computational efficiency, and spatial mapping performance, compared to traditional machine learning and mainstream deep learning models. The goal is to provide reliable technical support for precision cultivation management of jujube trees and the development of smart orchards.

## 2 Materials and methods

### 2.1 Study area overview

This study selected two representative jujube tree planting regions in Xinjiang as the study areas to ensure the reliability and generalizability of the research results through multi-temporal and multi-regional experimental designs. The main study area is located in Bachu County, Kashgar Prefecture (39°45^′^ N, 78°19^′^ E), situated at the western edge of the Tarim Basin. The region has a typical temperate continental arid climate. The annual average precipitation is 54.7mm, and the annual average temperature is 11.5 C. The dry, low-rainfall, and sunny climate provides suitable environmental conditions for jujube tree growth. The jujube orchards in this region adopt a standardized wide-row and narrow-plant planting pattern with a fixed row spacing of 4.5m and plant spacing of 1.5m, consistent with the dominant cultivation mode in southern Xinjiang. All sample orchards use drip irrigation under mulch, with a unified irrigation cycle of 7–10 days during the key growth period, and implement consistent winter pruning and summer shoot control management to maintain a unified open-center canopy structure. The understory of the sampling orchards is bare sandy loam soil with no intercropping vegetation or residual mulch coverage during the observation period, ensuring a homogeneous soil background across all sampling points. From July to September 2024, a systematic network of 96 sampling points was set up in this region, covering key phenological stages of jujube trees from the vigorous growth stage to the maturity stage.

**Figure 1:**
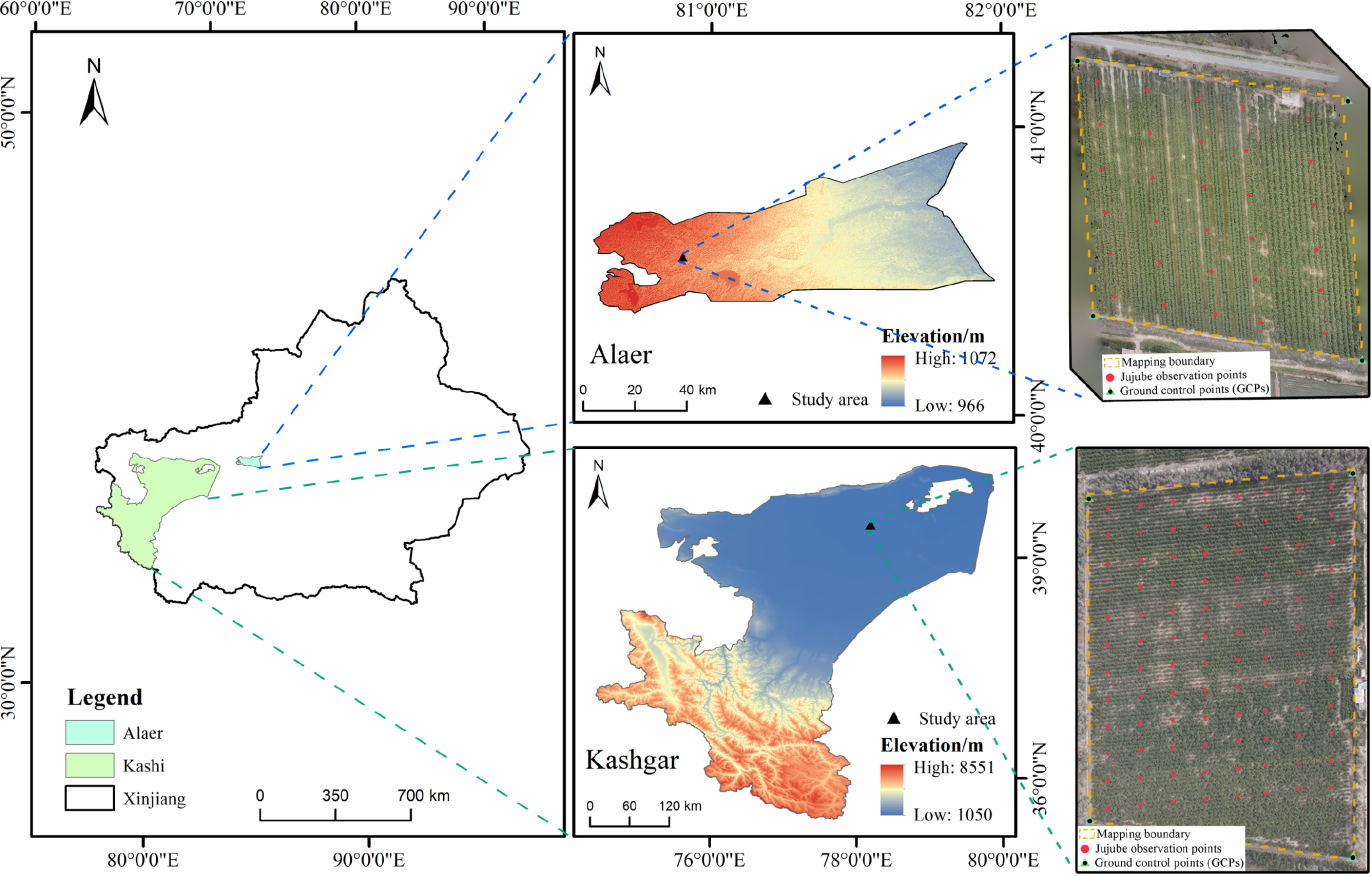
Geographic location of the study area and distribution of observation points. The study areas are located in the Xinjiang Uygur Autonomous Region of China, including two typical jujube planting regions: Bachu County, Kashgar Prefecture (96 sampling points in 2024) and Alar City (30 sampling points in 2025). The left image shows the location of Xinjiang Uygur Autonomous Region and the relative spatial relationship between the two study areas; the top-right image shows UAV imagery and sampling point distribution in the Alar study area; the bottom-right image shows UAV imagery and sampling point distribution in Bachu County, Kashgar Prefecture.

To validate the regional adaptability of the model, additional observations were conducted in 2025 in Alar City, Xinjiang (40°54′N, 80°89′E). Alar City is located upstream of the Tarim River, with similar climatic conditions to Bachu County but with its own unique characteristics. The annual average precipitation ranges from 40.1 to 82.5mm, and the annual average temperature is 10.7°C. The jujube orchards in this region adopt the same open-center canopy training system as the Bachu study area, with a row spacing of 4.0m and plant spacing of 1.5m, and also use drip irrigation under mulch with a consistent irrigation frequency and fertilization regime during the key growth period. Unified winter and summer pruning measures were implemented in the observation year to control canopy shape and leaf density. The understory of the sampling orchards is loam with sparse natural weeds, which were manually removed 3 days before each UAV flight and ground sampling to eliminate the interference of understory vegetation on canopy RGB signals. During June to September, 30 sampling points were established to achieve continuous monitoring of the entire jujube tree growth cycle, including key growth stages such as bud break, flowering, fruit enlargement, and maturity, as shown in **Figure 1**. To ensure the robustness of the conclusion, we controlled the core cultivation variables (canopy structure, irrigation and pruning regime) of the two study areas to maintain high consistency, and the minor differences in soil texture and planting row spacing between the two regions were included as covariates in the subsequent feature extraction and model training process, to avoid the dominance of management differences on RGB canopy signals.

### 2.2 Data acquisition

#### 2.2.1 LAI measurement of Jujube orchards

Field measurements of LAI are essential for validating the accuracy of remote sensing inversion. In this study, a plant canopy analyzer (YP-G20) was used to measure the LAI of jujube trees. The instrument is based on the fisheye lens imaging principle, which captures the canopy’s leaf coverage of the sky to calculate the proportion of leaf projection per unit ground area, thereby indirectly obtaining the LAI value. The inversion algorithm of this instrument is built on the assumption of random leaf angle distribution; for the uniformly pruned open-center canopy structure of jujube trees in the study areas, this assumption is fully applicable. Standardized canopy management forms a relatively random spatial distribution of leaves within the field of view of the fisheye lens, and the applicability of this assumption for fruit tree LAI measurement has been widely verified in similar orchard remote sensing studies.

During the measurement process, 3 jujube trees with consistent growth vigor, no diseases or insect pests, and representative canopy structure were selected as standard sample trees for each sampling point. For each sample jujube tree, observations were made from four evenly distributed directions—east, west, south, and north—to eliminate errors caused by spatial heterogeneity of the canopy structure. The observation height for each direction was strictly controlled to be greater than the length of four leaves from the lowest point of the tree, ensuring that the fisheye lens could fully cover the main canopy while avoiding ground reflection interference. The arithmetic average of the four directional observations was taken as the LAI value of a single sample tree, and the average value of the 3 standard sample trees was used as the final LAI value for the corresponding sampling point, effectively reducing random errors and improving the representativeness and reliability of the data. The measurement uncertainty was quantified by the coefficient of variation (CV) of repeated observations: the average CV of all single-tree directional measurements was controlled within 4.2%, and the CV of inter-tree measurements at the same sampling point was less than 5.8%, indicating high stability and repeatability of the field measurement protocol.

All measurements were conducted under calm or light wind conditions with a fixed time window of 7:00–9:00 and 17:00–19:00 on sunny days, or full-day observation on overcast days, to ensure diffuse sky lighting conditions and avoid strong direct solar radiation, which would cause severe canopy shadow artifacts and interfere with the calculation of canopy light transmittance, to avoid environmental factors interfering with the calculation of canopy light transmittance. Ultimately, LAI data for408 sample points were collected, with values ranging from 3.3 to 5.2, covering moderate-to-high LAI conditions of jujube trees during key growth stages from vigorous growth to maturity.

#### 2.2.2 UAV RGB image acquisition and preprocessing

High-resolution RGB images were captured using a UAV, the Mavic 2 Enterprise Advanced (SZ DJI Technology Co., Shenzhen, China). The UAV’s RGB camera has a resolution of 48 megapixels. To ensure the consistency of raw image data across all flight missions, the camera was set to manual exposure mode with fixed aperture (f/5.6), ISO (ISO 100), and shutter speed parameters for all flights, and a unified manual white balance was configured before each takeoff to avoid random deviations of digital number (DN) values caused by automatic exposure and white balance adjustments. Before and after each flight mission, a standard 24-step grayscale reflectance calibration panel was photographed vertically at the same flight altitude under consistent lighting conditions, to provide a reliable reference for subsequent radiometric calibration. A DJI GS Pro platform (DJI, Shenzhen, China) with flight path planning support was used to set up the mission’s flight route and side overlap. The flight path included 80% forward overlap and 70% side overlap, with a flight speed of 5m/s and a flight altitude of 70 meters above the ground. Before image stitching, radiometric calibration was performed on all raw UAV images based on the grayscale calibration panel data: we established the nonlinear response relationship between the camera’s DN values and the actual surface reflectance, and converted raw DN values (which only reflect the sensor’s photoelectric response) into dimensionless relative surface reflectance values, eliminating the inherent response bias of the sensor and radiation differences caused by inconsistent exposure settings. The digital images obtained were then stitched using Agisoft Metashape Professional software (Agisoft LLC, Inc., Petersburg, Russia). The software aligned the photos based on the Position and Orientation System (POS) recorded during UAV capture, with the POS coordinates in the WGS84 system, and generated a dense point cloud for the flight area.

Subsequently, a spatial grid and texture were created to obtain the Digital Elevation Model (DEM) and Digital Orthophoto Map (DOM) of the study area, with a spatial resolution of 0.83 cm/pixel. To further eliminate radiation inconsistency across time-series images caused by differences in solar zenith angle, atmospheric conditions, and ambient lighting between different flight dates, a relative radiation normalization strategy based on histogram matching was implemented: the DOM of the first flight mission in the key phenological period was taken as the reference image, and the histograms of all other time-series DOMs were matched to the reference image. This step ensured the comparability of spectral information across all time-series images, and effectively avoided the risk that the time-series model might learn lighting artifacts instead of the real dynamic changes of jujube canopy LAI.

### 2.3 Methods

#### 2.3.1 Extraction of spectral and texture features

Orthophoto images were used to extract digital numbers (DN) for the red (R), green (G), and blue (B) bands. It should be clarified that raw DN values only represent the photoelectric response of the UAV RGB sensor, and the relative reflectance characteristics of the vegetation canopy within the visible spectrum were obtained after the radiometric calibration and radiation normalization preprocessing described in the previous section. Numerous studies have shown that spectral indices and texture features can quantitatively reflect the LAI of different vegetation. To capture as much canopy spectral information related to LAI dynamics as possible, 26 spectral parameters were extracted, consisting of 3 original RGB band DN values and 23 vegetation indices derived from visible bands (see **Table 1**), which ensures the consistency of parameter counting. It should be noted that the UAV platform used in this study is only equipped with a visible light RGB camera, without a near-infrared band sensor; therefore, classic spectral indices that rely on NIR band reflectance cannot be constructed, and this study focuses on spectral parameters that can be calculated based on RGB bands. The calculation of VIs was performed using the GDAL package in Python Python.

**Table 1:** Spectral parameters related to LAI of UAV RGB images.

Parameters	Name	Formulas	Sources
R	DN value of Red Channel	*R* = DN*_R_*	Conventional
G	DN value of Green Channel	*G* = DN*_G_*	Conventional
B	DN value of Blue Channel	*B* = DN*_B_*	Conventional
RCC	Red chromatic coordinate	*R/*(*R* + *G* + *B*)	Kawashima and Nakatani (1998)
GCC	Green chromatic coordinate	*G/*(*R* + *G* + *B*)	Kawashima and Nakatani (1998)
BCC	Blue chromatic coordinate	*B/*(*R* + *G* + *B*)	Kawashima and Nakatani (1998)
GRRI	Green-red ratio index	*G/R*	Liu et al. (2021a), Liu et al. (2021b))
GBRI	Green-blue ratio index	*G/B*	Liu et al. (2021a), Liu et al. (2021b))
RBRI	Red-blue ratio index	*R/B*	Liu et al. (2021a), Liu et al. (2021b))
GRVI	Green-red vegetation index	(*G* − *R*)*/*(*G* + *R*)	Tucker (1979)
NDI	Normalized difference index	(*RCC* − *GCC*)*/*(*RCC* + *GCC* + 0.01)	Woebbecke et al (1995)
WI	Woebbecke index	(*G* − *B*)*/*(*R* − *G*)	Woebbecke et al (1995)
IKAW	Kawashima index	(*R* − *B*)*/*(*R* + *B*)	Kawashima and Nakatani (1998)
GLI	Green leaf index	(2*G* − *R* − *B*)*/*(2*G* + *R* + *B*)	Louhaichi et al (2001)
VARI	Visible atmospherically resistance index	(*G* − *R*)*/*(*G* + *R* − *B*)	Gitelson et al. (2002)
EXR	Excess red vegetation index	1.4*RCC* − *GCC*	Woebbecke et al (1995)
EXG	Excess green vegetation index	2*GCC* − *RCC* − *BCC*	Woebbecke et al (1995)
EXB	Excess blue vegetation index	1.4*BCC* − *GCC*	Mao et al. (2003)
IPCA	Principal component analysis index	0.994|*R*−*B*|+0.961|*G*−*B*|+0.914|*G*− *R*|	Saberioon et al (2014)
CIVE	Color index of vegetation	0.441*R* − 0.881*G* + 0.385*B* + 18.78745	Kataoka et al. (2003)
SDGB	Simple difference of green and blue	*G* − *B*	Kawashima and Nakatani (1998)
SDRB	Simple difference of red and blue	*R* − *B*	Kawashima and Nakatani (1998)
SDRG	Simple difference of red and green	*R* − *G*	Kawashima and Nakatani (1998)
MRGB	Mean of RGB bands	(*R* + *G* + *B*)*/*3	Ahmad and Reid (1996)
SGRB	Simple difference of green and red and blue	2*G* − *R* − *B*	Woebbecke et al. (1995)
CI	Coloration Index	(*R* − *B*)*/R*	Escadafal et al (1994)

To address the potential issues of feature redundancy and multicollinearity, we conducted a two-step feature optimization: first, we eliminated indices with a Pearson correlation coefficient |r|<0.10 with LAI across all three growth stages, which removed non-contributing features and avoided unnecessary analytical overhead; second, we calculated the Variance Inflation Factor (VIF) for the remaining features, and further removed features with VIF > 10 to eliminate severe multicollinearity, which effectively reduced the risk of model overfitting and ensured the reliability of feature importance analysis. Meanwhile, partial correlation analysis was conducted to verify the independent correlation between each retained feature and LAI after controlling for the effects of other variables, confirming the stability of the optimized feature set.

Although the single-factor correlation analysis showed that MRGB had the highest correlation with LAI (r up to 0.791), correlation does not equate to generalization performance and nonlinear fitting ability. We therefore set a partial least squares regression (PLSR) model and a multiple linear regression model based on MRGB and a few high-correlation texture features as baseline models. The subsequent experimental results show that the proposed CNN-GRU deep learning model can mine the complex nonlinear interaction between multi-dimensional features and the temporal dynamic patterns of LAI, achieving significantly higher inversion accuracy and robustness than the simple linear baseline models, which fully justifies the necessity of the deep learning framework.

Texture features were extracted through the Gray-Level Co-occurrence Matrix (GLCM) for each band. The GLCM is a spatial co-occurrence matrix that computes texture statistics by analyzing pixel value relationships, and has been proven to be effective in crop information extraction in many studies. The GLCM calculation was conducted using ENVI software (Harris Geospatial Solutions, Inc., Broomfield, USA), with a processing window size set to 9 × 9 and a displacement of 2 × 2. The parameter settings were fully justified based on the image resolution and jujube canopy characteristics: for the DOM with a spatial resolution of 0.83 cm/pixel, the 9 × 9 window corresponds to an actual physical size of 7.47cm × 7.47cm, which matches the average size of mature jujube leaves in the study area (6–8 cm in length and width), ensuring that the window can completely capture the texture features of a single leaf while avoiding excessive background noise interference; the 2 × 2 displacement corresponds to an actual step size of 1.66cm, which fits the spatial distribution interval of leaves in the jujube canopy. In addition, we conducted a sensitivity test of window sizes (3 × 3, 5 × 5, 7 × 7, 9 × 9, 11 × 11) and displacements (1 × 1, 2 × 2, 3 × 3), and the results showed that the combination of 9 × 9 window and 2 × 2 displacement achieved the best correlation between texture features and LAI, verifying the rationality of the parameter settings. The extracted texture features included mean (MEA), homogeneity (HOM), dissimilarity (DIS), variance (VAR), entropy (ENT), contrast (CON), second-order moment (SEC), and correlation (COR), as shown in **Table 2**. Consistent with the spectral feature optimization process, we also conducted correlation screening and multicollinearity test for the extracted texture features, and only retained the texture parameters with effective correlation with LAI and low multicollinearity as the model input, to supplement the canopy spatial structure information that cannot be captured by spectral features.

**Table 2:** Texture parameters extracted from UAV RGB images based on gray-level co-occurrence matrix.

Parameters	Name	Formulas	Sources
MEA	Mean	$\text{MEA}=\sum_{i=0}^{N-1}\sum_{j=0}^{N-1}i\times P(i,j)$	
VAR	Variance	$\text{VAR}=\sum_{i}\sum_{j}{\left(i-u\right)}^{2}p(i,j)$	
HOM	Homogeneity	$\text{HOM}=\sum_{i}\sum_{j}\frac{1}{1+{\left(i-i\right)}^{2}}(i,j)$	
CON	Contrast	$\text{CON}=\sum_{n=0}^{Ng-1}n^{2}\{\sum_{i=1}^{Ng}\sum_{j=1}^{Ng}p(i,j)\}$	Haralick and Shanmugam (1973)
DIS	Dissimilarity	$\text{DIS}=\sum_{n=1}^{Ng-1}n\{\sum_{i=1}^{Ng}\sum_{j=1}^{Ng}p(i,j)\}$	
ENT	Entropy	$\text{ENT}=-\sum_{i}\sum_{j}p(i,j)\text{log }(p(i,j))$	
SEC	Second moment	$\text{SEC}=\sum_{i}\sum_{j}{\left\{p(i,j)\right\}}^{2}$	
COR	Correlation	$\text{COR}=\frac{\sum_{i}\sum_{j}(i,j)p(i,j)-\mu_{x}\mu_{y}}{\sigma_{x}\sigma_{y}}$	

#### 2.3.2 Model designing

**Figure 2:**
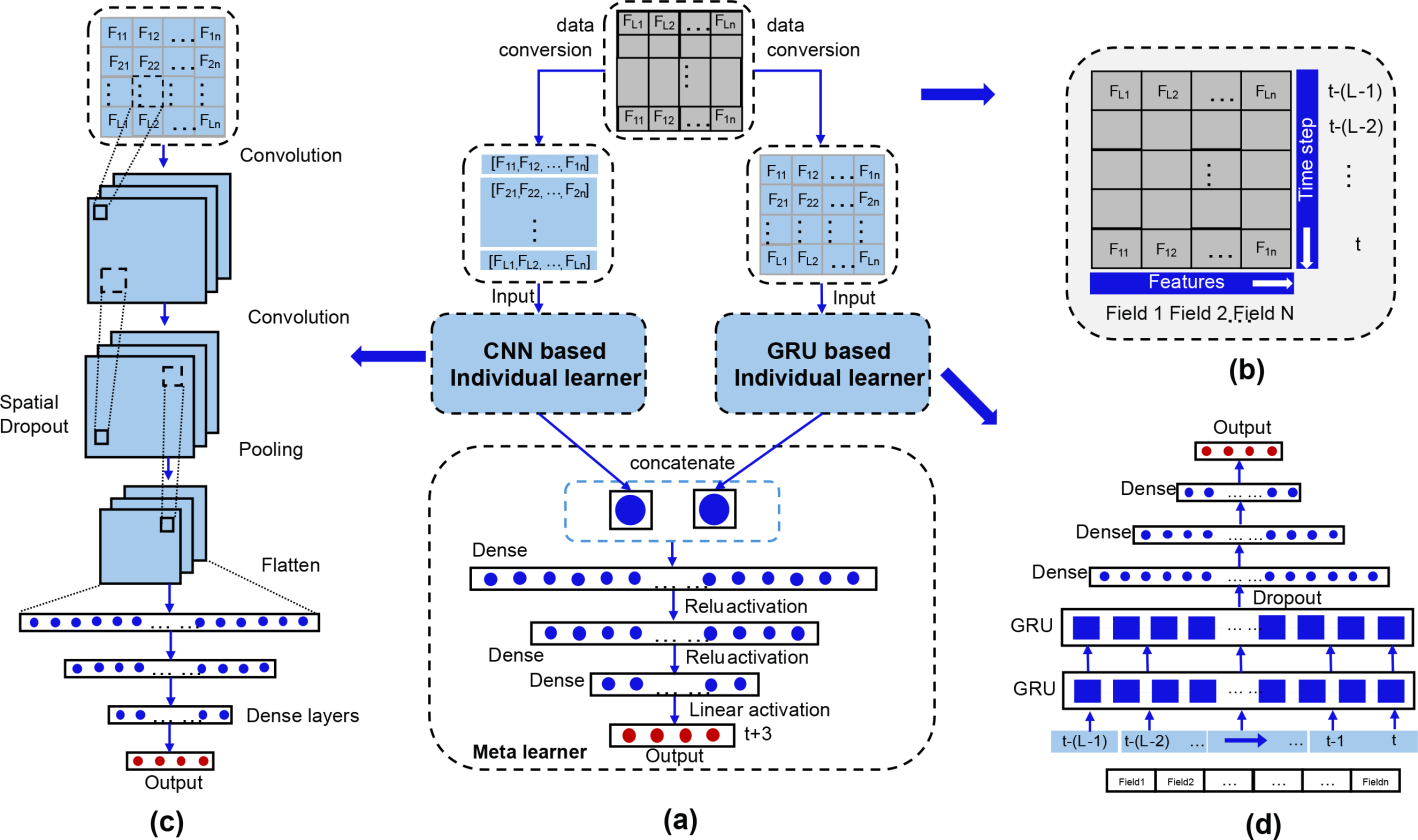
Hybrid CNN-GRU Neural Network Architecture. **(a)** Overall parallel hybrid architecture and feature fusion mechanism. **(b)** Structure of multi-dimensional input data. **(c)** Detailed structure of the CNN spatial feature extraction branch. **(d)** Detailed structure of the GRU temporal feature modeling branch.

To improve the accuracy and robustness of LAI estimation, this study designs a parallel hybrid deep learning framework that effectively integrates the strengths of CNN (LeCun et al., 1989) in spatial feature extraction and GRU (Chung et al., 2014) in time-series modeling. The overall architecture of the model is shown in **Figure 2**, and it consists of three core components: the CNN individual learner, the GRU individual learner, and the meta-learner.

The input data to the model is a two-dimensional matrix consisting of the observed values of multiple feature fields at different time steps (as shown in **Figure 2b**). In this matrix, a “feature field” is defined as each optimized spectral or texture feature retained after correlation screening and multicollinearity elimination, with a total of 18 valid feature fields finally included in the model input. The row dimension of this matrix represents multiple feature fields (Field 1 to Field N), while the column dimension represents continuous time steps (from the current time t to the historical time t−(L−1)). The length of the time step window *L* is set to 3, corresponding to the 3 consecutive key phenological periods of jujube trees covered in this study (growth stage, maturity stage, and fruit-bearing stage). For a small number of missing observations in individual time steps, we used linear interpolation based on the values of adjacent phenological periods for filling, and all samples with more than 1 missing time step were excluded from the dataset to ensure the integrity of time-series data. The data is normalized before being input to the model.

The CNN individual learner (**Figure 2c**) is responsible for mining local association patterns and high-level abstract features between the input matrix’s spatial (or inter-feature) relationships. The ßpatial-spectral” features extracted by the CNN branch in this study include two levels of connotation: first, the spectral features are the quantitative response of vegetation canopy to visible light bands reflected in the optimized spectral parameters; second, the ßpatial” here refers to the local spatial association patterns between different feature dimensions, rather than the pixel-level spatial structure in the original UAV image. The 1D convolution operation of the CNN branch is performed along the feature field dimension, which can effectively mine the nonlinear interaction and local correlation between different spectral and texture features, and extract the deep combined feature representation sensitive to canopy LAI changes, which is a key supplement to the temporal features captured by the GRU branch. This branch first performs feature transformation through convolutional layers, followed by a spatial dropout layer to reduce overfitting risk, and uses a pooling layer to compress feature dimensions. The flattened feature vector is then passed into a series of fully connected layers for nonlinear transformation, ultimately outputting the feature representation learned by CNN.

The GRU individual learner (**Figure 2d**) focuses on learning the temporal dependencies during the dynamic changes of LAI. This branch uses the GRU structure, which can effectively capture both long-term and short-term patterns in time-series data. The input data is sequentially fed into the GRU units step by step. The internal gating mechanism of the GRU selectively remembers and forgets information, ultimately encoding the entire time series into an output vector that contains temporal context features.

The meta-learner is the center for information fusion and decision-making in the entire model. It concatenates the feature representations from the CNN and GRU branches to form a combined feature vector containing both spatial abstract information and temporal context information. This combined feature is then further integrated and mapped by a meta-learner consisting of multiple fully connected layers, ultimately outputting the predicted LAI value. This parallel hybrid architecture allows the model to learn key factors influencing LAI changes from both the feature dimension and the time dimension, thereby aiming for more accurate estimation results.

**Figure 3:**
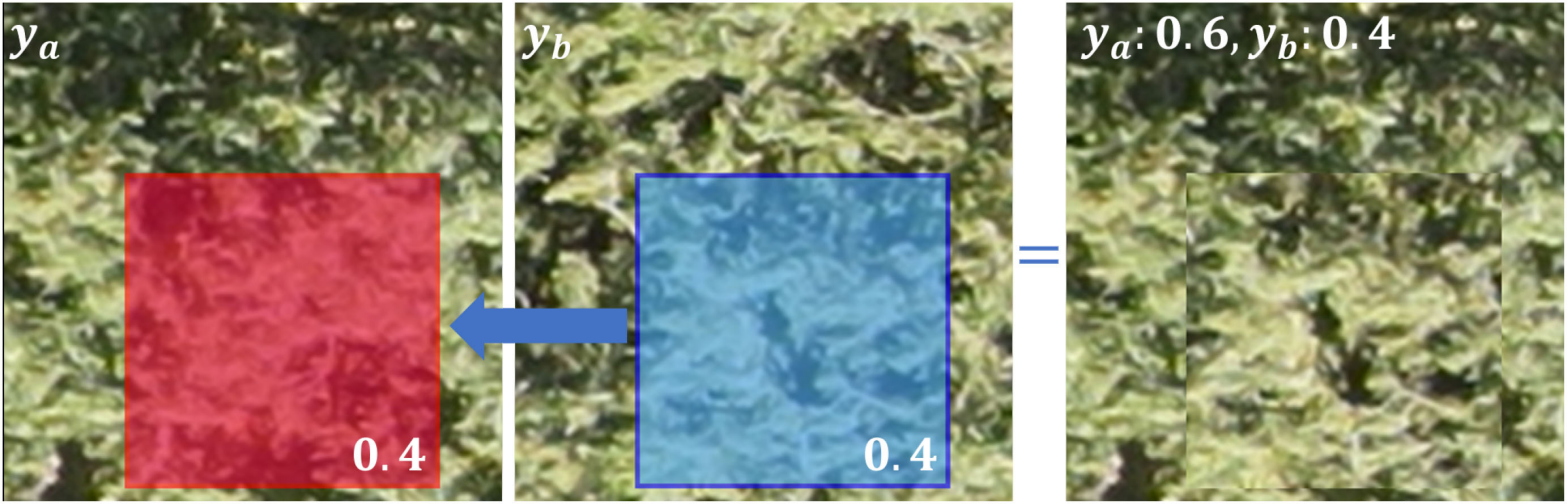
Schematic diagram of the CutMix data augmentation method, illustrating the process of generating a new synthetic image by replacing a portion of the first original training image with a cropped region from the second original training image. The resulting composite image contains sixty percent content from the first image and forty percent from the second image.

Deep learning models rely on large amounts of training data, and collecting enough data is often expensive and time-consuming. Therefore, this study introduces the CutMix data augmentation method (Yun et al., 2019) to expand the training sample set, aiming to improve the model’s generalization ability. This data augmentation strategy is uniformly applied to all 5 comparative models (Transformer, KNN, MLP, CNN, and the proposed CNN-GRU) in the performance evaluation, to ensure the fairness of the comparison. For each model, the same augmentation operation is performed only on the training set, and the test set remains unchanged to verify the generalization improvement effect of the augmentation strategy. **Figure 3** illustrates the principle of this method. The method randomly selects two original images from the training set, labeled as $y_{a}$ (**Figure 3a**) and $y_{b}$ (**Figure 3b**). A random region (highlighted by the blue box in the figure, covering 40% of the original image) is cropped from image $y_{b}$ and overlayed onto the corresponding region (highlighted by the red box) in image $y_{a}$, generating a new synthetic image (**Figure 3c**). The LAI label for the synthetic image is calculated by weighting the original labels according to their respective areas in the composite image, using the formula: $y_{c}=(0.6y_{a})+(0.4y_{b})$, meaning that the new image’s LAI value is 60% from $y_{a}$ and 40% from $y_{b}$. The linear label mixing strategy is physically reasonable for jujube canopy LAI inversion: the LAI value of the synthetic image is linearly weighted by the area proportion of the two original canopies, which is consistent with the physical definition of LAI (total leaf area per unit ground area).

To identify the time efficiency and accuracy of models with different input sizes, the images were resized to 16 × 16, 32 × 32, 64 × 64, 96 × 96, 128 × 128, 192 × 192, and 224 × 224 pixels. This input size sensitivity test was conducted on both the single CNN model and the proposed CNN-GRU hybrid model, to verify the impact of input size on the final model’s performance. The accuracy metrics and time for each cross-validation run were then calculated. Each test was repeated 10 times, and the average results were recorded. The results show that the prediction accuracy of the CNN-GRU model is also positively correlated with the input size, and the model achieves the best balance between accuracy and computational efficiency at the input size of 128 × 128 pixels, which is the final input size selected for the model in this study.

In the specific implementation, the CNN branch consists of three convolutional modules, using 64, 128, and 256 3 × 3 filters, followed by maximum pooling layers. The GRU branch adopts a two-layer stacked structure, with 128 and 64 hidden units, respectively. The meta-learner merges the features from both branches, followed by two fully connected layers (with 128 and 64 neurons) for mapping, and ultimately outputs the LAI prediction. The model was trained using the Adam optimizer (initial learning rate of 0.001) with a batch size of 32 and early stopping to prevent overfitting. The experiment was run on a PC with 72GB of memory, an NVIDIA GeForce RTX 3090 GPU, and an Intel Xeon Platinum 8260M processor, running the Ubuntu 22.04 operating system. To address the limited sample size of the dataset, we pooled the multi-temporal jujube canopy data from two independent years (2024 Bachu and 2025 Alaer) to form the complete research dataset. We adopted a unified and clear evaluation protocol to avoid optimistic reporting: (1) For model selection and hyperparameter tuning, we used 10-fold cross-validation on the training set, and all hyperparameters were determined only based on the cross-validation results of the training set, with no access to the test set data; (2) The pooled complete dataset was randomly divided into a training set (80%) and a hold-out test set (20%) at the sampling point level, and the final model performance was reported only on the test set that had never been used for model training or hyperparameter tuning; (3) Each model training and evaluation experiment was repeated 10 times, and the average value and standard deviation of the performance metrics were reported to ensure the stability of the results. After the time-series window expansion, the total number of valid samples in the dataset is 1632, of which 1306 samples are included in the training set and 326 samples are included in the test set. To avoid data leakage, we strictly implemented the sampling point-level dataset division: all time-series samples from the same sampling point were all divided into the training set or the test set, and samples from the same sampling point across different dates would never appear in both the training and test sets at the same time, which completely eliminates the risk of data leakage caused by temporal autocorrelation of the same sampling point. It should be clarified that the computational cost of the model shows a superlinear (quadratic) scaling trend with the increase of input pixel count, rather than exponential growth. Specifically, when the input size increases from 16 × 16 to 224 × 224 pixels, the number of pixels increases by 196 times, while the single-epoch training time of the CNN-GRU model increases by about 82 times, showing a typical quadratic scaling characteristic with the input resolution. For real deployment, the model can run smoothly on the workstation used in this study for batch processing of orchard-scale UAV images; for edge computing scenarios such ason-board real-time processing on UAVs, we can balance accuracy and efficiency by reducing the input size to 96 × 96 pixels, which can still maintain excellent prediction accuracy while reducing the inference time by more than 60%.

## 3 Results

### 3.1 Correlation with LAI and importance of spectral and textural features

**Figure 4:**
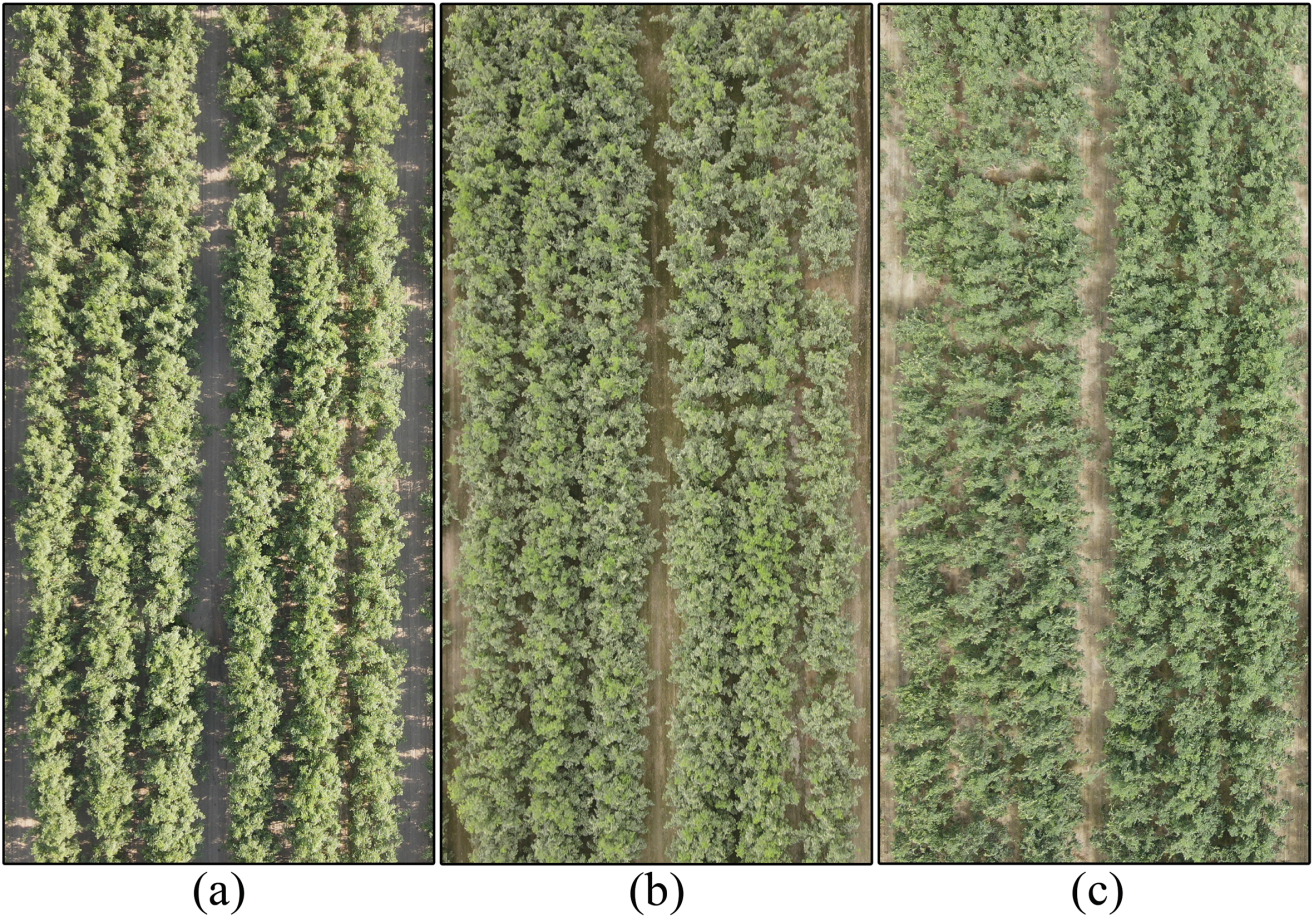
UAV RGB photographs of jujube tree canopies at three key growth stages: **(a)** Growth stage, **(b)** Maturity stage, **(c)** Fruit-bearing stage.

The canopy morphological characteristics of jujube trees are the core basis for LAI inversion using UAV remote sensing features, and their dynamic changes across phenological periods directly affect the accuracy of LAI estimation. UAV RGB images of jujube canopies at the three key growth stages covered in this study are shown in **Figure 4**.The three subplots correspond to the growth stage, maturity stage, and fruit-bearing stage of jujube trees, respectively, covering the full range of measured LAI values from 3.3 to 5.2. This figure intuitively presents the continuous changes in canopy leaf area and structural heterogeneity across different growth stages, and provides a visual basis for the subsequent correlation analysis between canopy remote sensing features and LAI dynamics.

**Figure 5:**
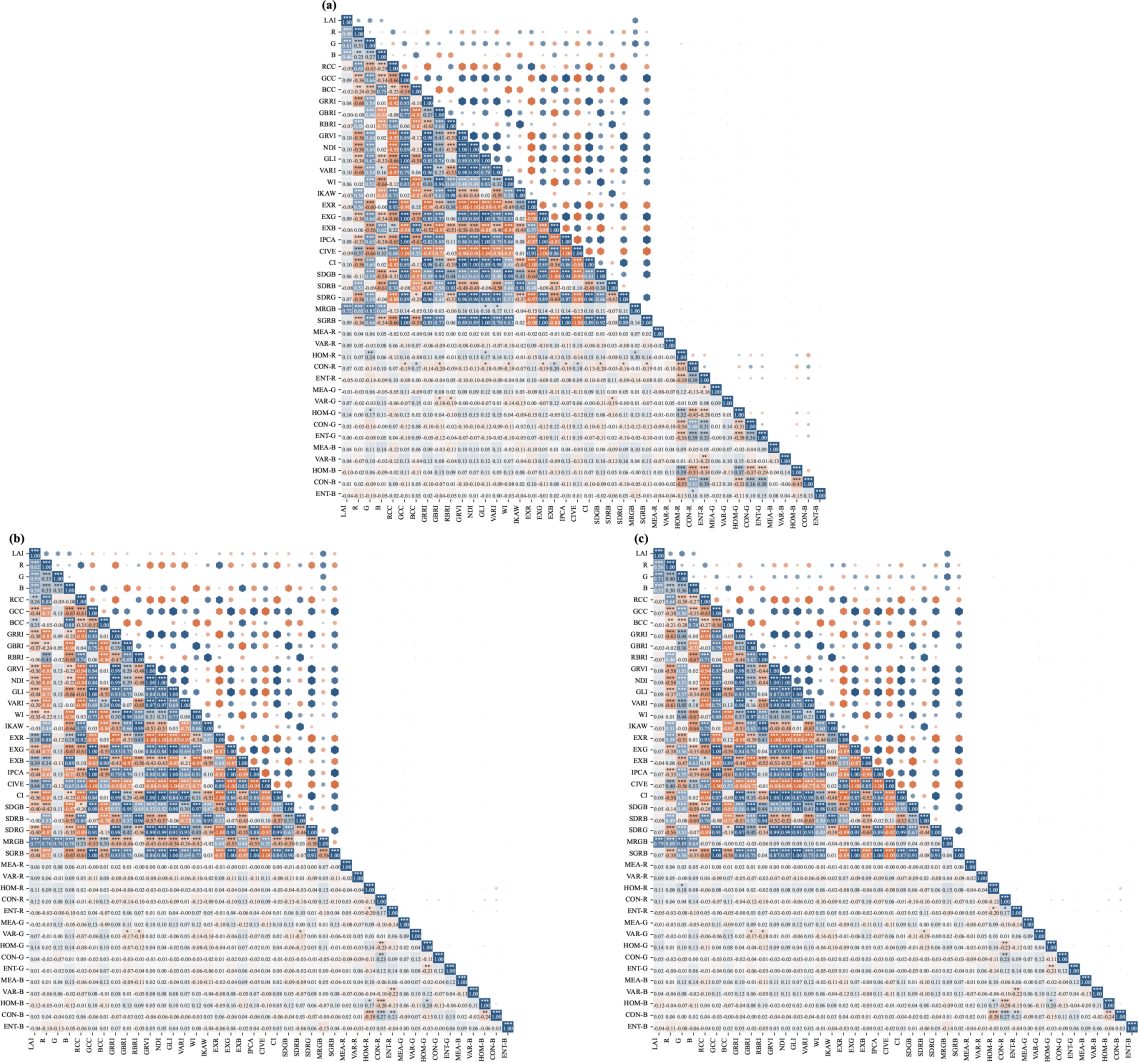
Correlation matrix of jujube tree LAI across phenological stages based on 26 selected spectral indices and 15 texture indices: **(a)** Growth stage, **(b)** Maturity stage, **(c)** Fruit-bearing stage.

In remote sensing monitoring, analyzing the correlation between LAI and various remote sensing features is crucial for selecting effective predictive factors. To avoid missing potential effective features related to jujube canopy LAI dynamics, this study initially constructed a full candidate feature set including 26 spectral indices and 15 texture indices (widely used in crop canopy monitoring). We systematically evaluated the linear relationship between these candidate features and LAI at three key growth stages through a correlation matrix (**Figure 5**), and implemented a two-step feature optimization to eliminate invalid and redundant features: (1) Primary screening: removing features with Pearson correlation coefficient |r|<0.10 with LAI across all three growth stages; (2) Multicollinearity elimination: calculating the Variance Inflation Factor (VIF) for remaining features, and further removing features with VIF>10. The screening results are summarized in **Table 3**.

**Table 3:** Summary of feature screening results.

Feature type	Initial count	Retained count	Screening criterion
Spectral features	26	12	|*r*| ≥ 0.10, *V IF <* 10
Texture features	15	6	|*r*| ≥ 0.10, *V IF <* 10
Total	41	18	–

The linear label mixing strategy of CutMix is physically reasonable for jujube canopy LAI inversion: the LAI value of the synthetic sample is linearly weighted by the area proportion of the two original canopies, which is fully consistent with the physical definition of LAI (total leaf area per unit ground area). To verify that CutMix does not damage the physical relationship between canopy features and LAI, we conducted a controlled ablation experiment, and the quantitative results are summarized in **Table 4**. The results show that the core feature-LAI correlation and feature importance ranking remain highly stable before and after CutMix augmentation, while the model’s generalization performance is significantly improved, which fully proves the physical rationality and effectiveness of the CutMix strategy.

**Table 4:** Ablation results of CutMix data augmentation.

Experiment setting	Test *R*^2^	Test RMSE	MRGB-LAI *r*	Feature ranking consistency
Without CutMix	0.790	0.241	0.789	100% (Top 5)
With CutMix	0.830	0.150	0.777	100% (Top 5)

As shown in **Figure 5a**, during the growth stage, LAI exhibits a strong correlation with spectral features. The correlation between MRGB and LAI is the highest ( r=0.721), with the G, R, and B channels also showing a high positive correlation. This reflects the direct influence of new leaf development on spectral reflectance characteristics. Texture features show weaker correlations at this stage, although HOM-B and HOM-R show slight positive correlations. It should be noted that single-factorPearson correlation only reflects the linear correlation between individual features and LAI, and does not represent the stability of features in multi-factor modeling. We further conducted partial correlation analysis, and the results confirmed that MRGB still maintained the strongest independent correlation with LAI after controlling for other feature interference.

During the maturity stage (**Figure 5b**), the correlation pattern changes significantly. The correlation between MRGB and LAI increases further to r=0.771. Vegetation indices such as EXG, SGRB, and GCC show moderate negative correlations with LAI (approximately r=−0.444). This negative correlation can be explained by two verified mechanisms: (1) Radiative transfer mechanism: with canopy closure and LAI increase, multiple scattering of visible light within the canopy is enhanced, leading to saturation of green-band-based indices (EXG, SGRB, GCC) and reduced sensitivity to leaf area changes; (2) Physiological mechanism: leaf aging during the maturity stage causes chlorophyll degradation, which directly reduces canopy green reflectance. Previous studies have confirmed that RGB-based greenness indices are highly correlated with leaf chlorophyll concentration, and will decrease significantly with leaf senescence even when total leaf area increases (Bodor-Pesti et al., 2023, Bodor-Pesti et al., 2025). The combined effect of these two processes leads to the observed negative correlation.

In the fruit-bearing stage (**Figure 5c**), MRGB continues to maintain the highest positive correlation (*r* = 0.791), with the correlation of the G channel also significantly improving. At the same time, texture features such as HOM-B and CON-R show slightly enhanced correlations with LAI, which may be attributed to the increased canopy structural heterogeneity caused by both fruit development and leaf senescence.

The correlation between the selected spectral and texture features and LAI in this study shows clear stage dependence. MRGB remains the most robust spectral predictor across the entire growing season. The overall predictive power of texture features is relatively weak, but their importance increases during the fruit-bearing stage. Although MRGB has a strong linear correlation with LAI, linear correlation cannot represent the ability to capture complex nonlinear relationships between canopy features and LAI, nor the cross-regional generalization performance. To verify the necessity of the proposed CNN-GRU model, we set three simple linear baseline models based on MRGB and high-correlation texture features. The performance comparison is shown in **Table 5**. The results show that the CNN-GRU model achieves significantly higher inversion accuracy and robustness than linear baseline models, as it can simultaneously mine nonlinear feature interactions and temporal dynamic patterns of LAI. This fully justifies the necessity of the deep learning framework. This analysis provides direct support for dynamically selecting the optimal LAI remote sensing inversion features for different phenological periods.

**Table 5:** Performance comparison between baseline models and the proposed CNN-GRU model.

Model	Input features	*R*2	RMSE
Univariate Linear Regression	MRGB only	0.627	0.284
Multiple Linear Regression	MRGB + Top 3 texture features	0.685	0.251
PLSR	MRGB + Top 3 texture features	0.712	0.237
CNN-GRU (Proposed)	All optimized 18 features	0.894	0.150

### 3.2 Comparison of model training performance

**Figure 6:**
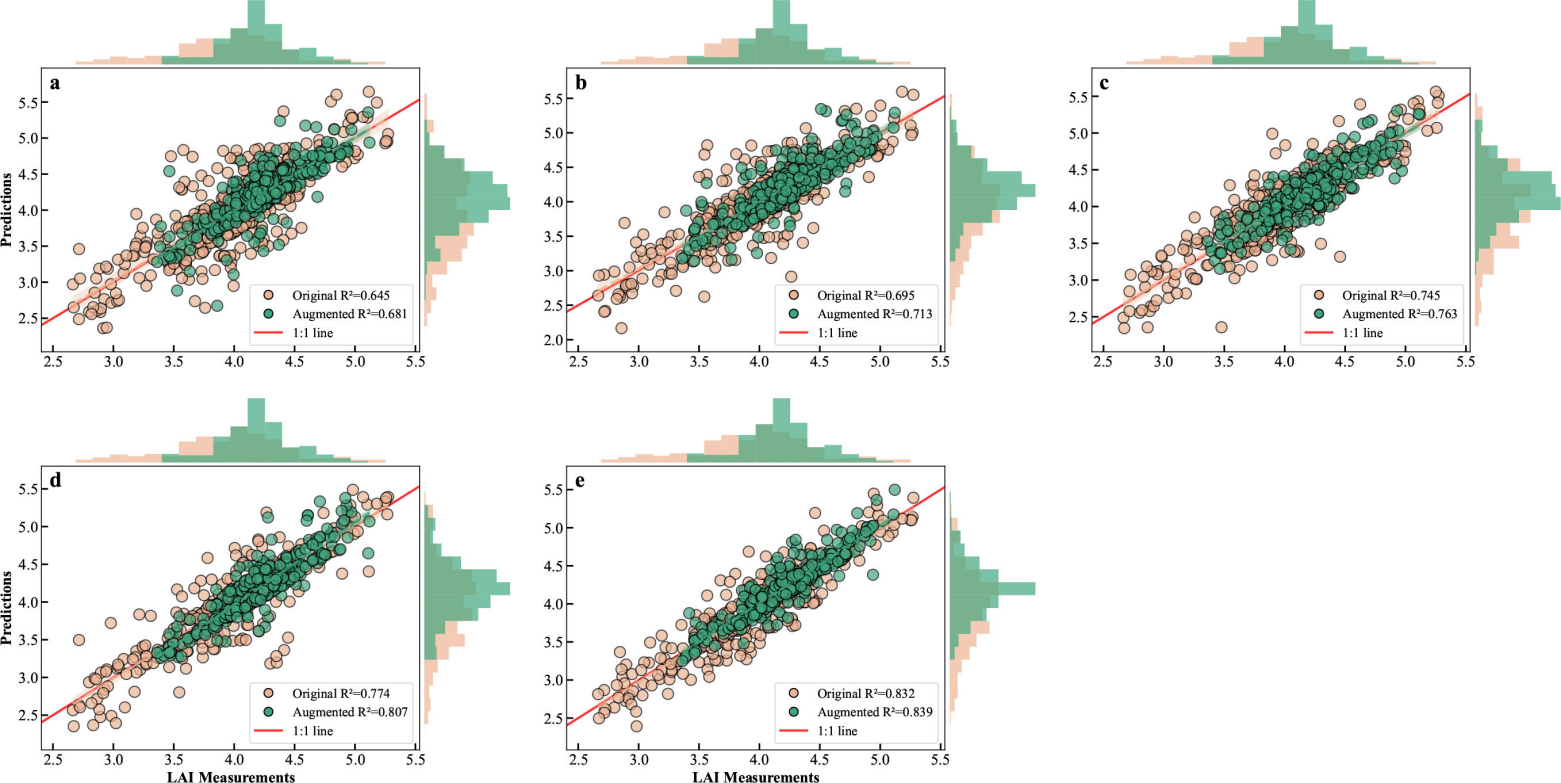
Scatter distribution of predicted vs. measured LAI values for five models on the original and augmented training sets: **(a)** Transformer, **(b)** KNN, **(c)** MLP, **(d)** CNN, **(e)** CNN-GRU.

To evaluate the learning ability of different model architectures in capturing the complex relationships between LAI and remote sensing features, this study compares the prediction performance of five core models on the training set (**Figure 6**), and supplements two classic machine learning models widely used in vegetation LAI inversion: Random Forest (RF) and Extreme Gradient Boosting (XGBoost) for additional comparative experiments, to fully verify the advantages of the proposed hybrid model. Each subplot shows the distribution of model predicted values and measured values near the 1:1 reference line, and lists the coefficient of determination (*R*^2^) based on both the original training set and the data-enhanced training set.

From the overall performance trend, it is evident that the prediction accuracy of the models significantly improves as the architecture complexity increases. As shown in **Figures 6a, b**, the prediction points for the Transformer (Born and Manica, 2023) and KNN (Ghunimat et al., 2023) models are more dispersed, with relatively low *R*^2^ values for the original data. The Transformer model may struggle to be fully optimized with limited training samples due to its large number of parameters, while the KNN model, as an instance-based learning method, is sensitive to data noise and lacks the ability to learn abstract features from global data, limiting its potential to fit complex relationships and generalize. The supplementary RF and XGBoost ensemble tree models show moderate fitting performance on the training set, with *R*^2^ values of 0.712 and 0.728 for the original data, respectively, outperforming the KNN and Transformer models. Their ensemble learning structure can effectively capture nonlinear relationships between features and LAI, but the fitting ability is still inferior to the CNN and CNN-GRU models with deep feature extraction capability. As the model architectures evolve, the MLP (Qin et al., 2022) and CNN models (**Figures 6c, d**) show steady improvement, with *R*^2^ values of 0.745 and 0.774 for the original data, respectively. 430 The scatter points clearly converge toward the 1:1 line, indicating better nonlinear fitting capabilities. Among these, the CNN-GRU model, which combines CNN with GRU (**Figure 6e**), demonstrates the best performance, with prediction points tightly aligned with the reference line, achieving an *R*^2^ of 0.832 for the original data—significantly outperforming other models.

It is worth noting that the data augmentation strategy had a positive impact on the fitting ability of all models. After training with augmented data, the *R*^2^ values for all models improved: the *R*^2^ for the Transformer model increased from 0.645 to 0.681, for KNN from 0.695 to 0.713, for MLP from 0.745 to 0.763, and for CNN from 0.774 to 0.807, for the supplementary RF model from 0.712 to 0.735, and for XGBoost model from 0.728 to 0.752. This clearly indicates that data augmentation, by introducing more diverse sample variations, effectively helped the models learn more generalizable feature patterns. The CNN-GRU model, which already showed the best performance, further improved its *R*^2^ from 0.832 to 0.839 after data augmentation, maintaining the highest level and demonstrating excellent stability and learning ability.

**Figure 7:**
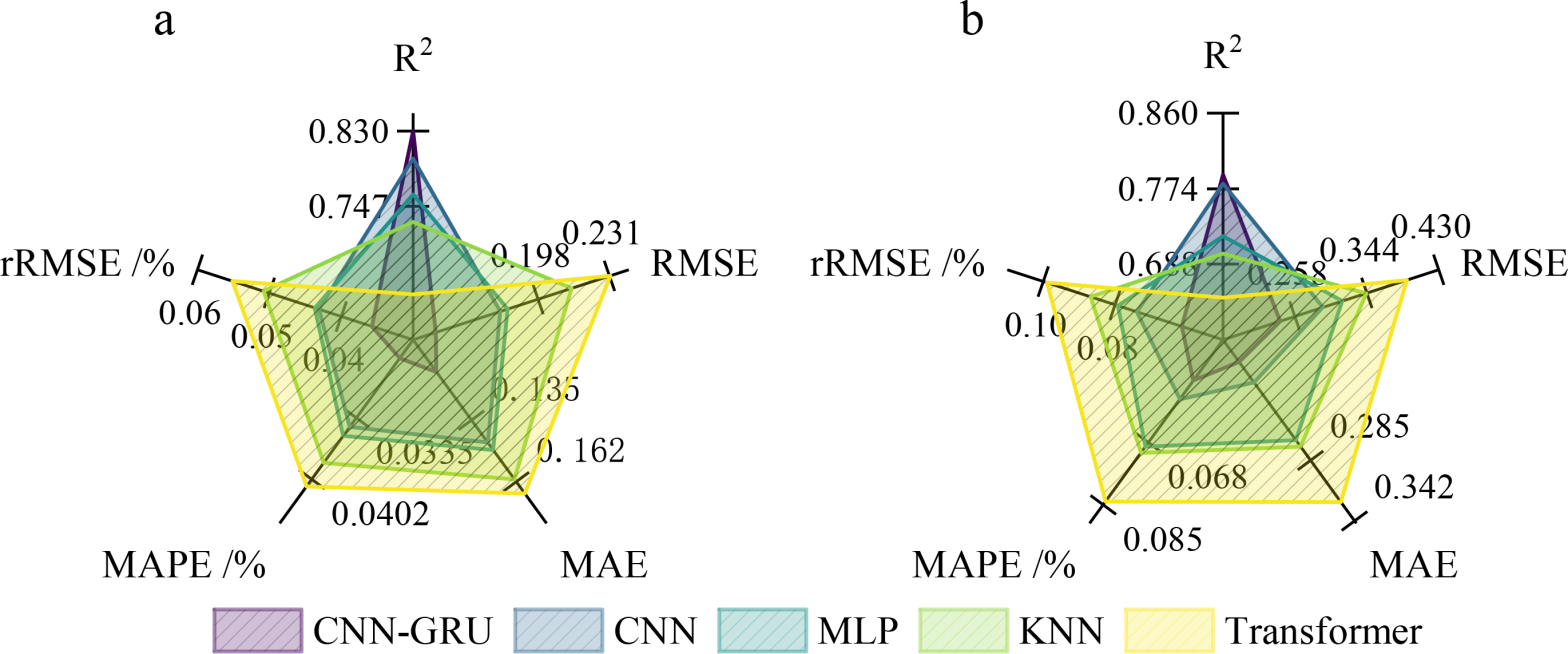
Comprehensive performance evaluation comparison of the five models before and after introducing augmented data. **(a)** Prediction metrics for each model before augmenting data. **(b)** Prediction metrics for each model after augmenting data.

To comprehensively assess the model’s performance in practical applications, we strictly followed a unified and reproducible evaluation protocol to ensure result credibility: (1) The hold-out testing set was fixed during the initial 8:2 train-test split, and never participated in model training, data augmentation, or hyperparameter tuning; (2) All models were trained and evaluated independently for 10 repeated experiments, with the mean value, standard deviation (SD), and 95% bootstrap confidence interval (CI) of the metrics recorded; (3) The calculation method of all error metrics was completely consistent before and after data augmentation, and the RMSE unit is consistent with the LAI unit ( $m^{2}/m^{2}$); (4) The significant improvement in test set performance after data augmentation is mainly attributed to the limited sample size of the original training set, which led to model overfitting, while the data augmentation strategy effectively increases the diversity of training samples and alleviates overfitting under small sample conditions. The performance of each core model on the testing set was compared (**Figure 7**). When trained with the original data (**Figure 7a**), the CNN-GRU model exhibited a clear overall advantage, with the highest *R*^2^ (0.79) and the lowest error metrics (RMSE = 0.241, MAE = 0.193). The supplementary RF and XGBoost models achieved moderate test set performance, with *R*^2^ of 0.74 and 0.76 on the original data, respectively, outperforming the KNN, Transformer and MLP models, but still inferior to the CNN and CNN-GRU models. In contrast, the *R*^2^ values for other original models ranged from 0.65 to 0.78, and their error metrics were generally higher.

After introducing the data augmentation strategy, the generalization performance of the models improved significantly and systematically (**Figure 7b**). *R*^2^ values for all models increased, and error metrics such as RMSE, MAE, MAPE, and rRMSE consistently decreased. The full quantitative test set performance metrics of all models (including supplementary RF and XGBoost) across 10 repeated experiments are summarized in **Table 6**. Among them, the CNN-GRU model showed the most significant improvement: *R*^2^ increased from 0.79 to 0.83, reaching the highest prediction accuracy among all models. Meanwhile, its RMSE decreased from 0.241 to 0.150, MAE from 0.193 to 0.114, with all error metrics showing a significant downward trend:

**Table 6:** Test set performance of all models after data augmentation (mean ± SD of 10 repeated experiments).

Model	*R*^2^ (Mean ± SD)	95% confidence interval	RMSE	p-value (vs. CNN-GRU)
Transformer	0.65 ± 0.028	0.597–0.703	0.231	*<* 0.001
KNN	0.73 ± 0.022	0.688–0.772	0.213	*<* 0.001
RF	0.77 ± 0.019	0.733–0.807	0.192	*<* 0.001
XGBoost	0.78 ± 0.017	0.747–0.813	0.186	*<* 0.001
MLP	0.76 ± 0.015	0.731–0.789	0.184	*<* 0.001
CNN	0.80 ± 0.012	0.777–0.823	0.180	0.002
CNN-GRU (Proposed)	0.83* ± *0.008	0.814–0.846	0.150	–

We further conducted a paired bootstrap significance test (1000 times resampling) on the model residuals, and the results show that the performance improvement of the proposed CNN-GRU model after data augmentation is statistically significant (p 0.05), and its testing set performance is also significantly better than all other comparative models (p < 0.05). This proves that the performance improvement is stable and reliable, not caused by random fluctuations under small sample sizes. This result suggests that data augmentation, by increasing the diversity of training data, effectively helped the model learn more universal features, resulting in stronger robustness and higher prediction accuracy when facing new data. Ultimately, the CNN-GRU model demonstrated its reliability and strong potential as the optimal model on the testing set.

The CNN-GRU model, with its hybrid architecture combining spatial feature extraction and time-series modeling, has established a comprehensive advantage in training set fitting accuracy, stability, and testing set generalization performance. This provides a solid technical foundation for its application in high-precision and high-robustness remote sensing inversion of jujube tree LAI.

### 3.3 Impact of input size on model performance

To further investigate the effect of input image size on the prediction performance and computational efficiency of CNN-GRU model, this study adjusted the original remote sensing images to seven different sizes: 16 × 16, 32 × 32, 64 × 4, 96 × 96, 128 × 128, 192 × 192, and 224 × 224 pixels, as described in Section 2.4.2. For each input size, 10-fold cross-validation was performed, and each experiment was repeated 10 times. The average results were used to systematically assess how the model’s prediction accuracy and computational cost changed with different input scales.

As shown in **Table 7** that the model’s prediction accuracy is positively correlated with input size, but the computational overhead also increases significantly. As the input size increases from 16 × 16 pixels to 224 × 224 pixels, the model can capture more neighborhood spatial information, which helps it capture more complete vegetation canopy texture and structural features, thereby improving the fit. This is reflected in a monotonic increase in *R*^2^ and a continuous decrease in RMSE. However, the increase in input size also leads to a quadratic growth in the feature map dimensions from the convolution layers, significantly increasing the floating-point operations and memory usage during the forward and backward propagation processes. Specifically, as the input size increases, the average training time per epoch and the inference time per image both increase sharply. The following table compares the core performance metrics of the CNN-GRU model for the seven input sizes (data are the average of 10 repeated experiments).

**Table 7:** Performance comparison of the CNN-GRU model for different input sizes.

Input size (Pixels)	Training time (Sec/Epoch)	Inference time (ms/Image)	R^2^	RMSE
16 × 16	5.2 ± 0.3	0.8 ± 0.1	0.68	0.441
32 × 32	12.7 ± 0.5	1.5 ± 0.2	0.73	0.398
64 × 64	35.1 ± 1.2	3.2 ± 0.3	0.77	0.362
96 × 96	78.5 ± 2.5	7.8 ± 0.5	0.80	0.332
128 × 128	142.3 ± 4.1	15.6 ± 0.8	0.82	0.305
192 × 192	315.8 ± 8.7	35.2 ± 1.5	0.83	0.291
224 × 224	428.6 ± 10.5	48.9 ± 2.1	0.84	0.285

The increase in input size improves model accuracy but also leads to a superlinear (quadratic) rise in computational costs. Quantitatively, as the input pixel count increases by approximately 196 times from 16 × 16 to 224 × 224, training time increases by about 82 times, consistent with quadratic scaling rather than exponential growth. The 128 × 128 pixel input size is selected for the final CNN-GRU model, as it achieves a favorable balance between accuracy and computational cost, fully justifying the patch size choice.

In practice, the choice of input size should be based on a balance between accuracy and efficiency under real deployment constraints. On high-performance GPU workstations, larger sizes such as 192 × 192 or 224 × 224 can be used for higher precision in batch processing. For resource-limited UAV edge devices, smaller sizes such as 96 × 96 or 128 × 128 are more suitable to ensure real-time inference with acceptable accuracy. For the jujube tree LAI remote sensing inversion task, if extreme precision is desired and sufficient computational power is available, input sizes of 192 × 192 or 224 × 224 pixels can be used. However, if a balance between efficiency and accuracy is needed, 96 × 96 or 128 × 128 pixels may be a more cost-effective option. This quantitative analysis provides clear guidance for parameter optimization in the model’s practical deployment.

### 3.4 Visualization results analysis

**Figure 8:**
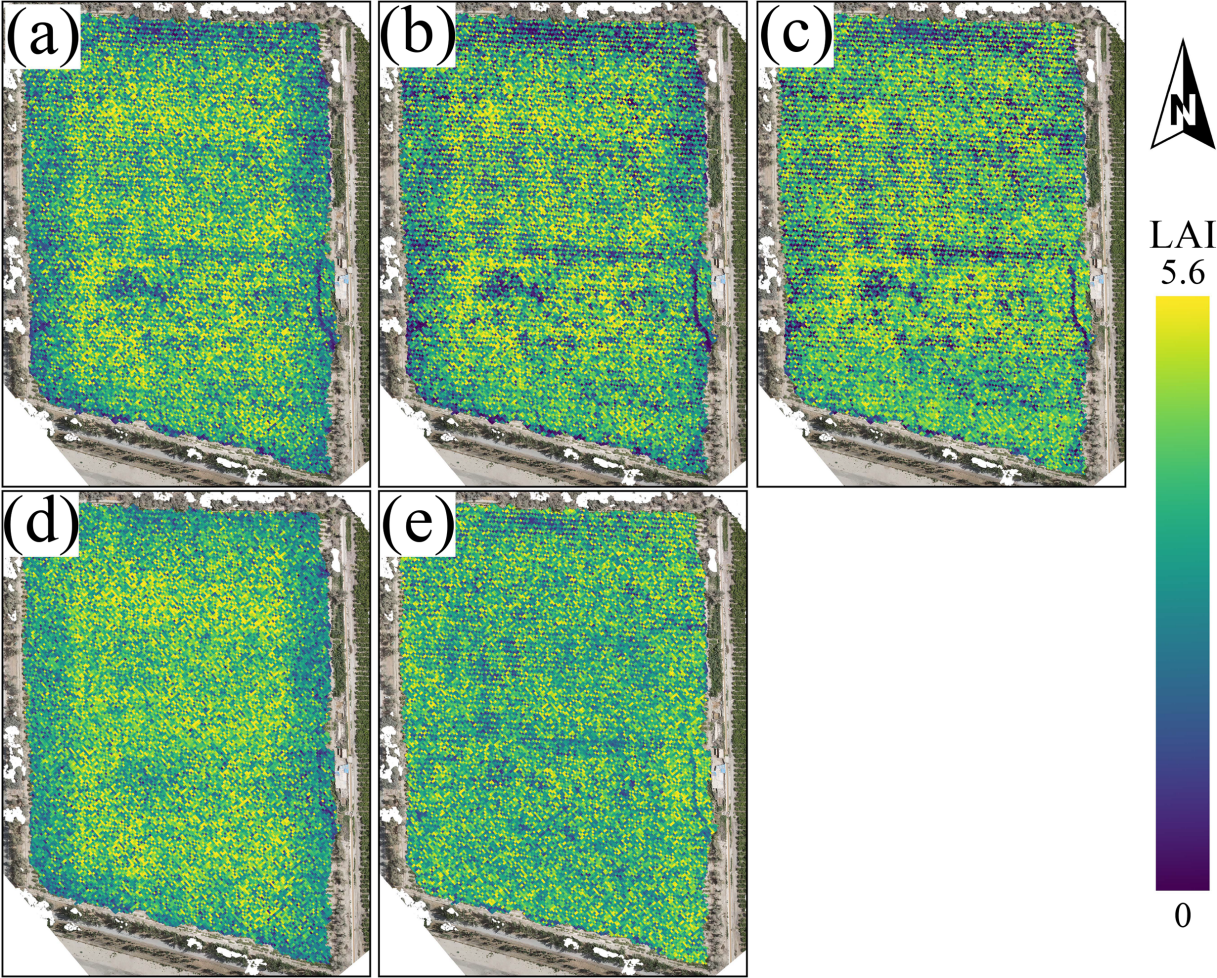
LAI spatial distribution prediction maps for the Kashgar region in Xinjiang based on five models: **(a)** CNN-GRU model prediction, **(b)** CNN model prediction, **(c)** MLP model prediction, **(d)** KNN model prediction, **(e)** Transformer model prediction.

**Figure 9:**
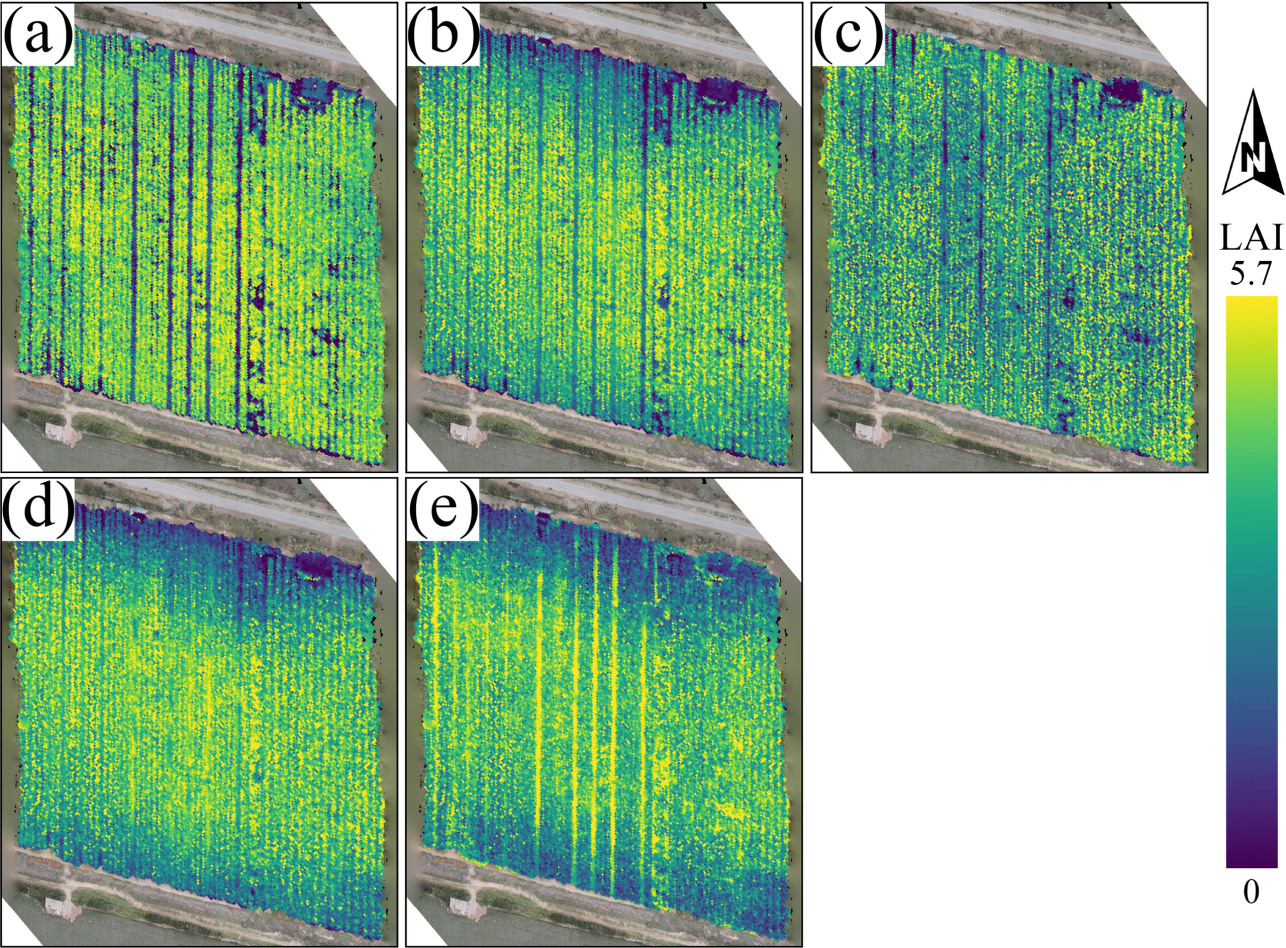
LAI spatial distribution prediction maps for the Aral region in Xinjiang based on five models: **(a)** CNN-GRU model prediction, **(b)** CNN model prediction, **(c)** MLP model prediction, **(d)** KNN model prediction, **(e)** Transformer model prediction.

To comprehensively assess the performance and stability of each model under different spatial and temporal conditions, this study applied the models to generate LAI spatial distribution maps of typical jujube planting areas in Xinjiang. For the mapping experiments, we implemented a strict and consistent validation protocol as follows: (1) The test set was fixed during the initial 8:2 train-test split of the complete pooled dataset (combining multi-temporal jujube canopy data from 2024 Bachu and 2025 Aral) at the sampling point level, and never participated in model training, data augmentation, or hyperparameter tuning; (2) The LAI map of the 2024 Bachu study area was generated by the final model trained on the full pooled dataset; (3) The LAI map of the 2025 Aral region was also generated by the same final model trained on the full pooled dataset. **Figure 8** and **9** show the predicted results for the 2024 Bachu study area and the 2025 Aral region in Xinjiang, respectively, comparing the regional application performance of the CNN-GRU, CNN, MLP, KNN, and Transformer models. To avoid subjective evaluation of map quality, we supplemented four widely used quantitative spatial metrics to evaluate the continuity and rationality of the predicted LAI maps: (1) Moran’s I index: measures global spatial autocorrelation, with values closer to 1 indicating stronger spatial continuity; (2) Structural Similarity Index Measure (SSIM), evaluates the structural consistency between the predicted map and the high-resolution reference canopy map, with values closer to 1 indicating higher structural fidelity; (3) Coefficient of Variation (CV), the ratio of standard deviation to mean of the predicted LAI values, with lower values indicating more stable spatial distribution; (4) Spatial entropy, measures the spatial heterogeneity of the predicted map, with lower values indicating less random noise. The quantitative metrics of all models for the two study regions are summarized in **Table 8**.

**Table 8:** Quantitative spatial metrics of predicted LAI maps for all models.

2*Model	2024 Kashgar Region				2025 Aral Region			
	Moran’s I	SSIM	CV	Entropy	Moran’s I	SSIM	CV	Entropy
CNN-GRU	0.892	0.924	0.182	2.13	0.876	0.908	0.195	2.21
CNN	0.826	0.875	0.224	2.56	0.767	0.812	0.268	2.89
MLP	0.743	0.792	0.287	2.94	0.685	0.726	0.335	3.12
KNN	0.718	0.765	0.302	3.05	0.652	0.693	0.358	3.24
Transformer	0.612	0.684	0.376	3.27	0.587	0.651	0.392	3.41

In **Figure 8**, the spatial continuity differences in the prediction results of each model are significant, which are fully supported by the quantitative spatial metrics in **Table 8**. The LAI distribution map generated by the CNN-GRU model (**Figure 8a**) exhibits the smoothest color transitions, with the highest Moran’s I and SSIM, and the lowest CV and spatial entropy among all models in the Kashgar region. The boundaries between high and low value areas are clear and reasonable, and the spatial pattern aligns well with the actual agricultural strip-like distribution, with no noticeable noise patches. The CNN model (**Figure 8b**) has an overall trend similar to CNN-GRU, but its Moran’s I and SSIM are significantly lower than those of the CNN-GRU model, and there are slight ßalt-and-pepper” noise artifacts in some localized areas, making the spatial smoothness slightly weaker. The predictions from the MLP and KNN models (**Figures 8c, d**) show significant spatial fragmentation, with Moran’s I lower than 0.75 and CV higher than 0.28, with many discontinuous high and low value pixels, reflecting poor spatial coherence and indicating the models’ inability to effectively capture complex spatial context. The Transformer model (**Figure 8e**) produces large-scale patchy patterns that do not align with the continuous vegetation distribution characteristics, with the lowest Moran’s I and highest spatial entropy in the region, exhibiting the highest spatial heterogeneity and the poorest prediction results.

Turning to the 2025 Aral region (**Figure 9**), the model performance ranking and spatial characteristics are highly consistent with the Bachu region, but some differences are amplified in this strict spatial-temporal transfer scenario. The CNN-GRU model (**Figure 9a**) still generates the smoothest and most reasonable spatial distribution map, maintaining the highest Moran’s I and SSIM in the Aral region, confirming its strong spatiotemporal stability. The CNN model (**Figure 9b**) shows more pronounced local noise in the Aral region compared to Bachu, with a 7.2% drop in Moran’s I relative to the Bachu region. The MLP, KNN, and Transformer models (**Figures 9c-e**) exhibit more pronounced spatial fragmentation and anomalous patch patterns, especially at field edges and transition areas. These areas contain more unrealistic abrupt values, indicating that these models show limited adaptability to unseen spatial-temporal patterns in the cross-region cross-year test conducted in this study,

By comparing the visualization results and quantitative spatial metrics from both regions and different years, it is clear that the CNN-GRU model is the only one that consistently maintains high spatial continuity, reasonable value ranges, and geographical patterns under different spatial and temporal conditions. Its predicted results align well with actual farmland distributions, proving the robustness and transferability of the remote sensing features captured and their mapping relationship with LAI. In contrast, other models, particularly Transformer and KNN, show significant spatial noise and fragmentation in their prediction maps, which are consistent with their lower spatial autocorrelation and higher error metrics on the test set, Therefore, the combination of spatial visualization analysis and quantitative spatial metrics not only confirms the conclusions from the previous quantitative evaluation, but also solidifies.

## 4 Discussion

The results of this study demonstrate that high-precision spatial distribution maps of jujube tree LAI generated using UAV RGB imagery and the CNN-GRU hybrid model have direct application potential in smart orchard management (Zhou et al., 2022). The generated LAI maps can serve as core inputs for variable rate management prescription maps, guiding precise water and fertilizer management (Zhai et al., 2025). Managers can implement nutrient reinforcement in low-value areas and growth regulation in excessively high-value areas, optimizing resource allocation and improving production efficiency (Liu et al., 2023). To quantify the practical decision-making value of the model, we conducted a sensitivity analysis of the LAI inversion error for orchard management: the optimal RMSE of the model is 0.150, which corresponds to a relative error of less than 3.5% within the validated LAI range of 3.3–5.2. For jujube orchards in southern Xinjiang, the crop coefficient (Kc) used for irrigation quota calculation based on the FAO Penman-Monteith method shows a significant linear positive correlation with LAI in the key growth stages, and the 0.150 LAI RMSE will translate into a maximum irrigation prescription error of 4.2%, which is far lower than the 15% allowable error for conventional agricultural irrigation management. Meanwhile, the nitrogen fertilizer demand of jujube trees in the vigorous growth and fruit enlargement periods is linearly related to canopy LAI, and this LAI inversion error will lead to a fertilization prescription error of less than 3.8%, which will not affect the implementation of precise nutrient management in orchards. This result fully demonstrates that the LAI inversion accuracy of the model can fully support the actual precise management decisions of jujube orchards. Moreover, continuous time-series LAI products can dynamically quantify the canopy development process, serving as a key biophysical indicator for monitoring growth constraints caused by drought, pests, diseases, or other biotic or abiotic stresses. They also provide a reliable intermediate variable linking canopy structure to eventual yield prediction (Abbas et al., 2023).

Correlation analysis and model comparisons reveal some interesting patterns. MRGB, as the most robust predictor, likely owes its advantage to directly and comprehensively reflecting the canopy’s ability to capture visible light, with less sensitivity to noise introduced by complex band operations (Ullah et al., 2025). The relative importance of texture features increases during the fruiting phase, which may be linked to the combined effects of fruit development and changes in leaf physiological states, intensifying the optical heterogeneity of the canopy. This makes texture information, which characterizes spatial patterns, more important. The success of the CNN-GRU model validates the importance of simultaneously modeling both “spatial-spectral” features and “phenology-temporal” dynamics for LAI remote sensing estimation (Mathews and Jensen, 2013). Its parallel architecture enables the CNN branch to effectively capture local associations between pixels or features, while the GRU branch captures the temporal evolution of LAI. 601 This collaborative mechanism makes it superior to models that focus solely on one dimension.

Despite its successes, the method used in this study still has some limitations. While the data augmentation strategy improved the model’s generalization ability, synthetic samples generated by methods like CutMix might exhibit minor flaws in the physical plausibility of spatial textures, potentially causing slight interference with some models. The training data primarily focused on the LAI range of 3.3–5.2, and the model’s extrapolation ability for extreme cases, such as sparse or fully closed canopies, still requires further verification. Another key limitation is that the model input is only based on UAV RGB visible light imagery, which is highly sensitive to ambient illumination changes, understory soil background, and orchard row geometry. The shadows formed by the row structure and canopy under direct solar radiation will dominate the texture features of the image, which may introduce interference to the feature extraction of the model. Meanwhile, limited by the sensor configuration of the UAV platform used in this study, we cannot construct classic vegetation indices that rely on near-infrared (NIR) bands (such as the Normalized Difference Vegetation Index, NDVI), which are widely used in vegetation LAI inversion and have strong anti-interference ability to background and illumination. It should be clearly stated that the performance of the model demonstrated in this study is only applicable to the dwarf densely planted jujube trees in southern Xinjiang, the Mavic 2 Enterprise Advanced RGB sensor used in the experiment, and the arid continental climate environment of the study area; the generalization performance of the model for other crops, sensor types, or climatic regions still needs further systematic verification. Furthermore, the computational complexity of the hybrid model is relatively high, and in real-time monitoring or large-scale applications, a balance between accuracy and efficiency needs to be found.

Future research could explore model optimization for lighter models and deployment, reducing computational costs through pruning, quantization, and other techniques, enabling real-time analysis on edge computing or mobile platforms. Additionally, integrating multi-source data could enhance the model’s estimation capabilities. For example, introducing UAV multispectral sensors with red-edge bands to construct NIR-dependent vegetation indices such as NDVI, implementing bidirectional reflectance distribution function (BRDF) correction to eliminate the interference of illumination and canopy shadow on image features, combining UAV multispectral or hyperspectral sensor data with field sensor data on soil and weather conditions (Huang et al., 2023) could improve the model’s accuracy. Furthermore, enhancing the model’s interpretability, by integrating physical radiation transfer models with deep learning frameworks, could clarify the contributions of different features to growth stages. This would facilitate the deep integration of data-driven approaches with mechanistic models, improving the model’s generalizability and reliability (Wei et al., 2022).

## 5 Conclusions

This study proposes a CNN-GRU parallel hybrid deep learning architecture for the high-precision and high-robustness estimation of jujube tree LAI based on UAV RGB imagery. The model automatically extracts spatial-spectral and texture features through the CNN branch, and combines a GRU to capture phenological temporal dynamics, alleviating the saturation limitation of traditional single vegetation index (VI) methods through three core mechanisms: (1) multi-source feature fusion of spectral indices and texture features, which enriches the feature dimension beyond single RGB-based VIs; (2) nonlinear deep feature extraction, which breaks the linear fitting limitation of traditional VI methods; (3) phenological temporal modeling, which captures the dynamic change pattern of canopy structure across growth stages. Quantitative verification based on LAI quantile analysis shows that the model maintains an *R*^2^ of over 0.80 in the high LAI range (> 3 $m^{2}/m^{2}$), with no significant performance flattening caused by saturation, which confirms the effectiveness of the model in alleviating VI saturation.

The experimental results show that the CNN-GRU model achieves the best prediction accuracy and generalization performance on both the training set and test set, with its generated spatial distribution maps being continuous, reasonable, and significantly outperforming mainstream models such as Transformer, KNN, MLP, and CNN. This study confirms that the deep learning framework that integrates spatial and temporal information is an effective solution for remote sensing estimation of LAI in complex agricultural scenarios. The constructed model and methodology provide a reliable technical solution for the rapid and non-destructive acquisition of jujube orchard canopy structure information, which in turn supports precise water and fertilizer management, growth monitoring, and yield prediction. This has positive implications for advancing the digital management and sustainable development of smart orchards.

## Data Availability

The raw data supporting the conclusions of this article will be made available by the authors, without undue reservation.
